# Expanding the chemical diversity of M13 bacteriophage

**DOI:** 10.3389/fmicb.2022.961093

**Published:** 2022-08-08

**Authors:** Grace L. Allen, Ashley K. Grahn, Katerina Kourentzi, Richard C. Willson, Sean Waldrop, Jiantao Guo, Brian K. Kay

**Affiliations:** ^1^Tango Biosciences, Inc., Chicago, IL, United States; ^2^Department of Chemical and Biomolecular Engineering, University of Houston, Houston, TX, United States; ^3^Department of Chemistry, University of Nebraska at Lincoln, Lincoln, NE, United States

**Keywords:** bioconjugation, phage-display, stop codon suppression, peptide, cyclization, cross-linking, combinatorial peptide libraries, antibody fragments

## Abstract

Bacteriophage M13 virions are very stable nanoparticles that can be modified by chemical and genetic methods. The capsid proteins can be functionalized in a variety of chemical reactions without loss of particle integrity. In addition, Genetic Code Expansion (GCE) permits the introduction of non-canonical amino acids (ncAAs) into displayed peptides and proteins. The incorporation of ncAAs into phage libraries has led to the discovery of high-affinity binders with low nanomolar dissociation constant (*K*_D_) values that can potentially serve as inhibitors. This article reviews how bioconjugation and the incorporation of ncAAs during translation have expanded the chemistry of peptides and proteins displayed by M13 virions for a variety of purposes.

## Introduction

Since the seminal work of Professor George Smith (Smith, 1985) in displaying a protein fragment on the surface of M13 bacteriophage, a large number of peptides and proteins have been displayed for antibody discovery, protein engineering, and mapping protein-protein interactions. While phage-display is a prize-worthy technique (Smith, 2019), it has generally been limited in chemistry to the canonical set of 20 amino acids of L-chirality. Both chemical modification and genetic methods have been used to expand the types of functional groups displayed on the surface of virions.

**FIGURE 1:**
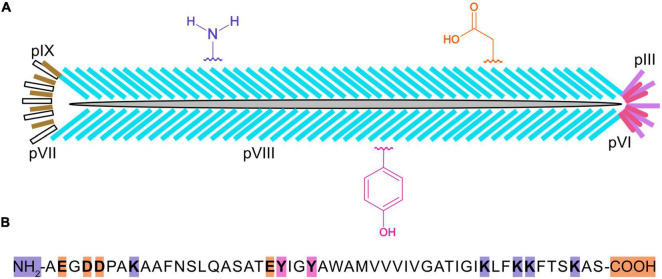
Cartoon of an M13 bacteriophage virion, three types of chemically reactive groups, and primary structure of the major capsid protein, pVIII. Each virus particle **(A)** is 900 nanometers long and 7 nanometers wide and contains one single-stranded, circular DNA molecule, and five copies of pIX (yellow), pVII (white), pIII (purple), and pVI (pink), and ∼2,700 copies of pVIII (blue). Chemical groups that are available for conjugation include the α-amino groups on the N-terminus and ε-amino groups of lysines (K) of pVIII, the carboxylate groups at the C-terminus and aspartic (D) and glutamic (E) acids, and the phenol groups of tyrosine (Y) are shown in violet, orange, and fushia, respectively. The primary structure for mature pVIII **(B)** is shown with the N-terminus, C-terminus, and residues theoretically capable of being chemically modified highlighted in color. It should be noted that the lysines in the anchoring region (KLFKKFTSKAS) of pVIII may not be sterically accessible for chemical modification.

Chemically modified virions have been used in a variety of applications (Mohan and Weiss, 2016), such as lateral flow assays (Hagström et al., 2015; Kim et al., 2015, 2017), biosensors (Moon et al., 2019), nanomaterials (Petrenko, 2018), nanomedicine (Ulfo et al., 2022), and batteries (Lee et al., 2009). In M13 bacteriophage (Figure 1), there are ∼2,700 copies of the major capsid protein, pVIII, which makes it a desirable target for bulk chemical modification of virions (Kehoe and Kay, 2005). The major capsid protein is 50 amino acids long and it contains a number of residues with functional groups suitable for bioconjugation, such as amines, carboxylic acids, and phenols (Carmody et al., 2021). The reactivity of these functional groups is dependent on steric accessibility, ionization state, and solvent conditions. For example, the ε-amino group of the lysine at position 8 can be derivatized with glutaraldehyde or N-hydroxysuccinimide esters (NHS-esters) to attach fluorescent dyes, biotin, drugs, DNA, enzymes, or gold nanoparticles (Carmody et al., 2021), although the N-terminus of pVIII is preferentially targeted because of its higher solvent accessibility and lower pK_a_ value (Li et al., 2010). While pVIII lacks cysteines, thiolation of primary amines with 2-Iminothiolane (Traut’s Reagent) generates sulfhydryl groups that can be coupled to maleimide-activated antibodies or enzymes. Moreover, ε-amino groups are reactive toward aldehydes that are easily generated by mild periodate-mediated oxidation of the sugars on antibodies or horseradish peroxidase (HRP), enabling favorable conjugation directed away from binding sites and active sites of antibodies or HRP, respectively (Adhikari et al., 2013, 2015). In another scheme, using 4-formyl succinimidyl benzoate, amine groups are converted to aromatic aldehydes that readily react with hydrazide derivatized DNA under mild conditions (Domaille et al., 2013). Carboxylate groups at the C-terminus and within aspartic (D) and glutamic (E) acids of virion coat proteins can be activated with a carbodiimide crosslinker [e.g., EDC (1-ethyl-3-(3-(dimethylamino) propyl)-carbodiimide hydrochloride)]. It is also possible to incorporate a methionine analog, L-azidohomoalanine (Urquhart et al., 2016), in strains of *Escherichia coli* that are auxotrophic for methionine and produce virions displaying hundreds of azide groups for downstream chemical conjugation.

Peptides and proteins that have been displayed on the surface of virions have also been the target of chemical or enzymatic modification. One can engineer a free cysteine (Junutula et al., 2008) for bioconjugation or, alternatively, disulfides in a displayed peptide, which can be reduced and then reacted with a cross-bridging molecule (i.e., linchpin), thereby creating macrocyclic or bicyclic peptides for the purpose of discovering novel peptide ligands of target proteins (Ng and Derda, 2016; Deyle et al., 2017; Chen et al., 2021; Ekanayake et al., 2021). Tyrosine (Y) in pVIII can be selectively activated by laccase to produce a free radical species that can be conjugated to acrylates (Vignali et al., 2018). Virions that have been engineered to display the AviTag (Scholle et al., 2004) or sortase tag (Hess et al., 2012) can be biotinylated by BirA or ligated to a variety of labeled peptides with sortase, respectively. Promising directions in the future will be to exploit the toolbox of enzymes capable of protein ligation (Nuijens et al., 2019; Weeks and Wells, 2020) and the SpyTag/SpyCatcher system (Keeble and Howarth, 2020) to build novel virion structures.

**FIGURE 2:**
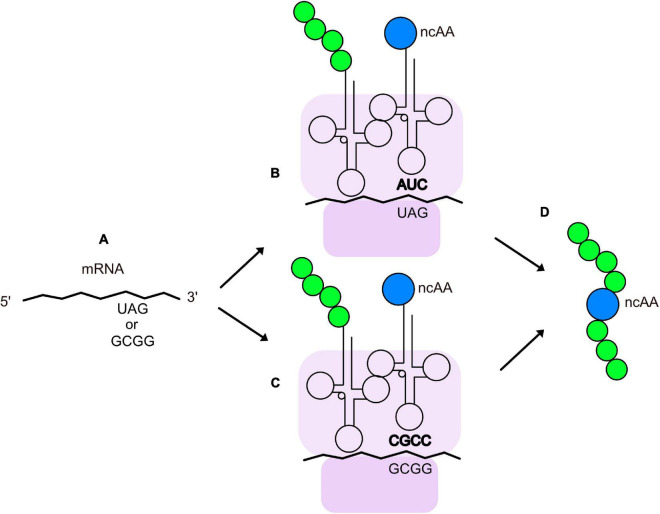
Insertion of ncAAs into peptides or proteins by GCE. **(A)** An mRNA containing an engineered quadruplet or stop codon for insertion of an ncAA at a designated location in a protein. During translation, the ribosome (purple) decodes the engineered stop codon **(B)** or quadruplet codon **(C)** with a tRNA charged with the ncAA (blue). The tRNA anticodon is shown in bold and the growing polypeptide chain is shown in green. **(D)** Peptide or protein with an ncAA at the desired location.

Genetic methods have been separately applied to expanding the chemical space of virions. They are largely based on the pioneering work of Professor Peter Schultz’s research group (Liu et al., 1997; Xiao and Schultz, 2016; Young and Schultz, 2018) on recoding the amber codon (TAG) so that it is recognized by a mutant, suppressor tRNA molecule that is charged with an non-canonical amino acid (ncAA). Figure 2 illustrates the basics of tRNA suppression. One first uses molecular biology techniques to engineer a TAG mutation at a site in the coding region of a protein where the ncAA is desired. When the gene is transcribed, the UAG codon is recognized as a stop codon by the ribosome, thereby terminating translation and yielding a truncated protein; however, if a tRNA has an anti-codon (CUA) that can base pair with the UAG codon in an mRNA, the amino acid attached to the 3’ end of the tRNA will form a peptide bond with the nascent peptide in the ribosome, allowing translation to continue and yield a full-length protein. The introduced ncAA can be any that is compatible with charging by an aminoacyl-tRNA synthetase (aaRS). The amino acid pocket of the pyrrolysyl-tRNA synthetase has been shown to be remarkably malleable to engineering recognition and charging of its cognate tRNA with diverse ncAAs (Wan et al., 2014; Tharp et al., 2018). Stop codon suppression has also been achieved with opal (UGA) (Anderson and Schultz, 2003) and ochre (UAA) (Italia et al., 2019) codons. In addition to suppression of stop codons, an analogous method with quadruplet codons (Magliery et al., 2001; Anderson and Schultz, 2003; Anderson et al., 2004; Neumann et al., 2010; Niu et al., 2013; Wang et al., 2014) has been developed (Figure 2). Mutations in both the aaRS and tRNA, as well as the use of engineered bacterial hosts (Chatterjee et al., 2014), have enhanced suppression efficiency of a variety of quadruplet codons (Guo and Niu, 2022). To date, >200 different ncAAs have been introduced into different proteins by this technique (Xiao and Schultz, 2016), which has been termed Genetic Code Expansion (GCE). Some of GCE’s applications have been to probe the structure and function of proteins, alter their redox potential, introduce fluorophores, infrared, and spin label probes, encode post-translational modifications, and create sites for site-specific bioconjugation. Several recent reviews of how GCE has been used to modify proteins expressed in bacteria, yeast, and mammalian cells can be found elsewhere (Young and Schultz, 2018; Chung et al., 2020; Nikić-Spiegel, 2020; Manandhar et al., 2021; Ros et al., 2021; Shandell et al., 2021; Sanders et al., 2022).

**FIGURE 3:**
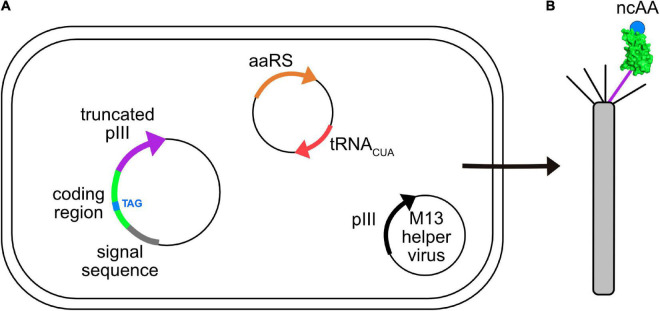
Incorporation of ncAAs into phage-displayed constructs. **(A)** *E. coli* cell showing three genomes: phagemid with suppressible mutation (TAG, blue) in the coding region (green) of a protein fused to a truncated form of capsid protein pIII (purple), a plasmid encoding an orthogonal aaRS/tRNA_CUA_ pair (orange/red), and an M13 helper virus (i.e., K07) encoding full length pIII (black). Each of these genomes carry a different antibiotic resistance marker to allow for selective growth of bacterial cells containing all three. **(B)** Virions secreted from such cells contain both wild type (black) and recombinant (purple) pIII capsid proteins, the latter of which display the protein of interest (green) incorporating the desired ncAA (blue).

GCE has been applied to phage-display. Figure 3 shows an *E. coli* cell containing three circular genomes: a plasmid encoding the orthogonal tRNA and cognate aaRS, a phagemid that carries a truncated form of capsid protein III (pIII), which is fused to the coding region of the displayed peptide or protein containing the suppressible amber codon, and an M13 helper virus. The helper virus encodes 10 proteins necessary for viral replication and assembly; it also contains a mutation that leads to preferential packaging of the phagemid genome over the helper virus genome. Secreted virions will display the recombinant peptide or protein and ncAA, only if suppression is successful. The culture medium is supplemented with the ncAA, where it enters the cell and is used by the engineered aaRS to charge the suppressor tRNA. Table 1 lists some examples of publications describing the incorporation of ncAAs into virions, which are also summarized in more detail below.

**TABLE 1:** Examples of GCE involving M13 virions in the literature.

Peptide or protein displayed on M13 virions	ncAA incorporated through GCE	Outcome	Reference
Combinatorial peptide library	Selenocysteine	Proof-of-principle experiment	Sandman and Noren, 2000
10-mer peptide epitope	Gln with Asp	Directed evolution of aminoacyl-tRNA synthetase	Pastrnak and Schultz, 2001
Short peptide	O-methyl-tyrosine p-azidophenylalanine p-acetylphenylalanine p-benzoylphenylalanine 3-(2-naphthyl)alanine	Proof-of-principle experiments demonstrated fluorescence labeling	Tian et al., 2004
scFv (TAG codon at position 111 of V_H_ CDR3)	Sulfotyrosine para-acetyl-phenylalanine bipyridyl-alanine 4-borono-phenylalanine	Affinity selection of a sulfotyrosine-containing antibodies that can bind to gp120	Liu et al., 2008
scFv with six random NNK codons in V_H_ CDR3	p-boronophenylalanine	Affinity selection of an scFv capable of forming a covalent bond to a sugar	Liu et al., 2009
CX_6_C, where X = NNK	Bipyridylalanine	Affinity selection of cyclic Ni^2+^ and Zn^2+^ binding peptides	Day et al., 2013
Five residues in the N-terminal finger of zif268 were randomized to include both canonical amino acids and Bpy-Ala	(2,2’-bipyridin-5-yl)alanine (Bpy-Ala)	DNA and Fe(II) binding domain	Kang et al., 2014
Peptide	Selenocysteine	Covalent attachment to five adenosine receptor ligands and activation of cell signaling pathways with decorated virions	Beech et al., 2015
CX_6_Z	*N*^ϵ^-acryloyl-lysine	Affinity selection of cyclic peptide ligands and inhibitor for TEV protease	Wang et al., 2019
scFv with TAG and quadruplet codons at five different positions	*N*^ϵ^-[((2-methylcycloprop-2-en-1-yl)methoxy)carbonyl]-l-lysine p-propargyloxy-l-phenyl-alanine	Dual labeling of scFv on virions	Oller-Salvia and Chin, 2019
CX_6_Z	four phenylalanine derivatives *N*^ϵ^-butyryl-lysine *N*^ϵ^crotonyl-lysine	Affinity selection of cyclic peptide ligands and inhibitors for Sirtuin 2 and TEV protease	Tharp et al., 2020
ZX_6_C	O-(2-bromoethyl)-tyrosine	Affinity selection of cyclic peptide ligands to streptavidin, Kelch-like ECH-associated protein 1 (Keap1), and Sonic Hedgehog (Shh)	Owens et al., 2020

C = cysteine.

X = NNK codons, where N is A, C, G, or T and K is G or T.1

Z = ncAA.

The earliest experiments of engineered replacement of amino acids with M13 bacteriophage took advantage of its life cycle properties. Scientists at New England Biolabs inserted the opal (TGA) stop codon and a downstream selenocysteine insertion sequence (SECIS) upstream of the signal sequence in pIII (Sandman and Noren, 2000). Viral secretion was dependent on the addition of selenium to the culture medium and the incorporation of selenocysteine was confirmed by chemical reactivity. The efficiency of incorporation was influenced by the choice of nucleotide downstream of the opal codon, although the frequency of TGG revertants, encoding tryptophan, was high. Later, the authors (Beech et al., 2015) utilized the selenocysteine-displaying virions to chemically attach five different adenosine receptor ligands and successfully demonstrated that the pentavalently decorated virions could activate the adenosine A1 receptor, a G protein-coupled receptor (GPCR), of cultured cells. An early example of utilizing stop codon suppression to control the replacement of an amino acid was that of the Schultz group (Pastrnak and Schultz, 2001). They introduced an amber codon *in lieu* of the asparagine (N) in a 10-mer peptide sequence (PASTTNKDKL) at the N-terminus of pIII of a phage genome. Virions were propagated in an *E. coli* strain that carried the supE mutation, which encodes a suppressor tRNA that is charged with asparagine. The virions were then used to infect a second bacterial strain that carried a plasmid encoding a yeast tRNA and its cognate aaRS that inserted N into the 10-mer peptide. Only virions secreted by this strain bound well to a monoclonal antibody that had been generated against the 10-mer peptide, demonstrating successful suppression of the stop codon. This proof-of-concept experiment set the stage for engineering aaRSs that could charge their cognate tRNA with ncAAs and suppress amber codons inserted into pIII of virions. Phage-assisted continuous evolution (PACE), phage-assisted non-continuous evolution (PANCE), and phage- and robotic-assisted near-continuous evolution (PRANCE) have enabled rapid laboratory evolution of orthogonal aaRSs over hundreds of generations of mutation, selection, and replication (Esvelt et al., 2011; Bryson et al., 2017; Miller et al., 2020; DeBenedictis et al., 2021, 2022; Fischer et al., 2022). As coat protein pIII is essential for phage, this selection scheme directly links the production and titer of virions with the efficiency of stop codon suppression and incorporation of the desired ncAA in a stop codon inserted into gene III.

The Schultz group was among the first to incorporate ncAAs into displayed peptides and proteins. A variety of ncAAs have been incorporated into peptides permitting bioconjugation (Tian et al., 2004), binding to metal ions (Day et al., 2013), and replacing Zn(II) with Fe(II) for DNA binding domains (Kang et al., 2014). Non-canonical amino acids have also been incorporated into human single-chain variable fragments (scFvs) displayed on virions. In fact, a sulfotyrosine has been incorporated into an scFv that contributed to binding to HIV glycoprotein 120 (Liu et al., 2008). The electrophilic amino acid *p*-boronophenylalanine has also been successfully incorporated into an scFv, where it can cross-link to a sugar (Liu et al., 2009).

**FIGURE 4:**
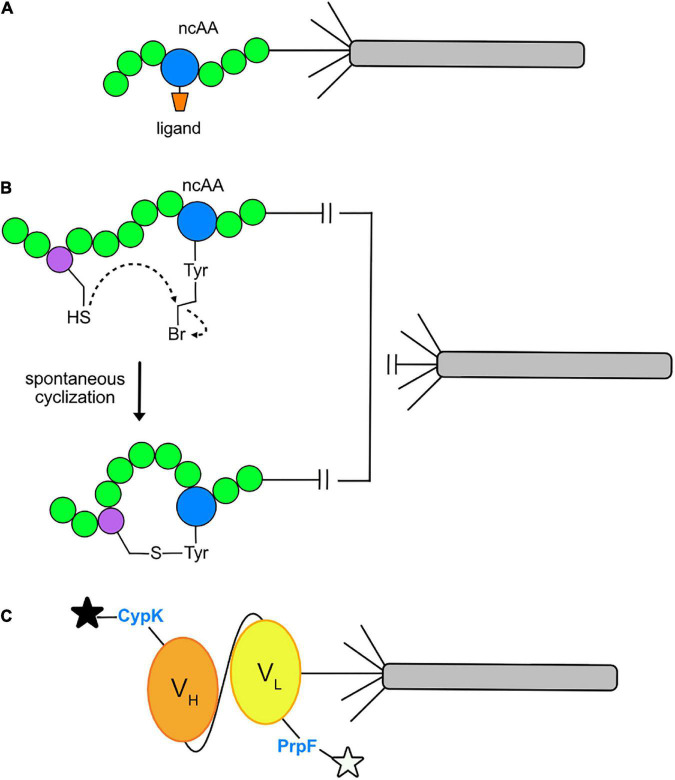
Current applications of GCE in phage display. **(A)** Conjugation of a ligand to an ncAA allows direct binding to the active site of a target enzyme. A ligand (orange) may be conjugated to an ncAA (blue) positioned within a peptide (green) and displayed on pIII of an M13 virion. After multiple rounds of phage-display affinity selections to the target protein, a consensus amino acid sequence flanking the ligand conjugated ncAA will be revealed. The final product is an optimized sequence with strong affinity for the target protein. **(B)** Macrocycle formation via spontaneous cyclization. The cysteine-reactive ncAA O2bey (O-(2-bromoethyl)-tyrosine, blue) spontaneously cyclizes with a cysteine residue (purple) to form a macrocycle that is displayed on pIII of an M13 virion. **(C)** Dual fluorophore labeling of a phage-displayed protein. Orthogonal incorporation of the ncAAs PrpF (*p*-propargyloxy-phenylalanine) and CypK (cyclopropene derivative of lysine) into the variable light chain (V_L_, yellow) and variable heavy chain (V_H_, orange) regions of a single-chain variable fragment (scFv) of a recombinant antibody. This allows for dual labeling with azide- and tetrazine-fluorophores, respectively, as depicted by the black and white stars.

Insertion of ncAAs into phage-displayed peptides offers a number of experimental opportunities. First, it can increase the chemical diversity of the combinatorial peptide libraries for the purpose of identifying novel peptide ligands to a target protein. Second, the ncAA can serve as a site for bioconjugation of ligands that already bind to a target, such as an enzyme, for the purpose of discovering a peptide inhibitor (Figure 4A). For example, in a recent report (Tharp et al., 2020), N^ε^ -butyryl-L-lysine (BuK), thiobutyryl (tBuK), and thiomyristoyl (tMyK) analogs were introduced in a combinatorial 11-mer library for the discovery of inhibitors of the NAD-dependent deacetylase, Sirtuin 2 (Sirt2). The ability to genetically encode post-translational modifications by GCE should prove useful for identifying inhibitors of various enzymes and cellular binding proteins in the future.

Recently, GCE has also been used to incorporate ncAAs for the purpose of cyclizing phage-displayed peptides. This effort represents an alternative route for cyclizing phage-displayed peptides to generate macrocycles with novel structures and the potential to inhibit protein-protein interactions in therapeutic applications (Deyle et al., 2017). In the original approach (Heinis et al., 2009), a library of peptides with three fixed cysteines was reduced and then reacted with a trifunctional compound, termed a “linchpin,” thereby creating bicyclic peptides. GCE has been used for the same purpose: researchers have built libraries of virions displaying CX_6_Z (Wang et al., 2019; Tharp et al., 2020) and ZX_6_C (Owens et al., 2020), where X represents an amino acid encoded by NNK codons and Z is an ncAA encoded by the TAG codon that is capable of reacting with the adjacent cysteine residue to form a macrocycle (Figure 4B). These libraries were screened by affinity selection and yielded nanomolar inhibitors of Tobacco Etch Virus (TEV) protease (Wang et al., 2019), Sirt2 (Tharp et al., 2020), and Kelch-like ECH-associated protein 1 (Keap1) and Sonic Hedgehog (Shh) (Owens et al., 2020). The cyclized peptides all showed stronger affinity to their targets than their linear counterparts. It will be exciting to see how this approach unfolds over time. Perhaps covalent inhibitors can be discovered by incorporating ncAAs capable of cross-linking with a target as reported elsewhere (Chen et al., 2021).

GCE has been used to site-specifically dual-fluorophore-label proteins displayed on the surface of M13 virions (Figure 4C). Suppression of amber and quadruplet codons facilitated the insertion of N^ε–^[((2-methylcycloprop-2-en1-yl)methoxy)carbonyl]-l-lysine (CypK) and *p*-propargyloxy-l-phenyl-alanine (PrpF) into separate sites of a phage-displayed anti-Her2 scFv (Oller-Salvia and Chin, 2019). The orthogonal *Methanococus janaaschii* (*Mj*) tyrosyl-trRNA synthetase (*Mj*TyrRs)/tRNA_CUA_ pair was used to incorporate PrpF, which was labeled with an azide-fluorophore. The pyrrolysyl-tRNA synthetase (PylRS)/tRNA orthogonal pair was used to incorporate CypK, which was labeled with a tetrazine-fluorophore. By exploring a variety of variables, the authors were able to achieve near wild-type display levels of the scFv for dual labeled, phage-displayed proteins. This system allows for mutually orthogonal and site-specific, dual-labeling of a protein in a one-pot reaction.

**FIGURE 5:**
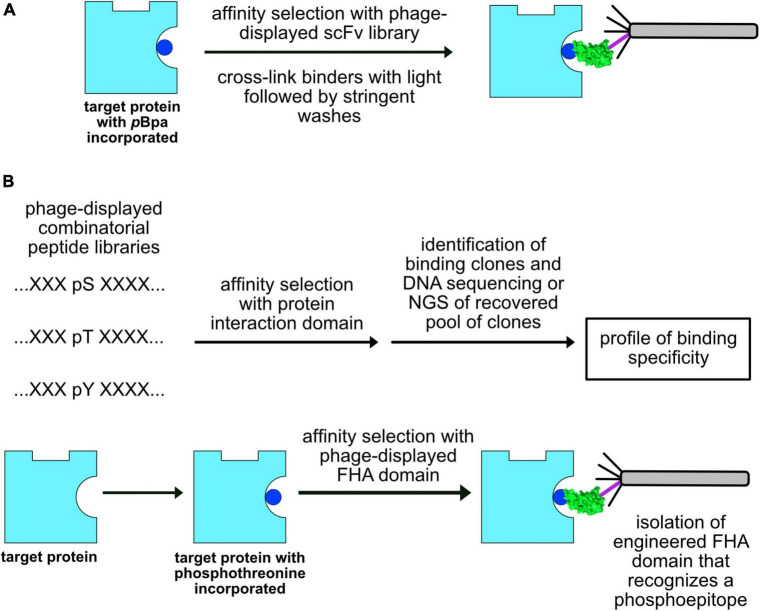
Future applications of GCE in phage display. **(A)** Epitope steering. If the target protein (cyan) has incorporated a photoactivatable, crosslinking ncAA (*p*Bpa, blue), recovery of virions can be biased toward those that bind to the labeled epitope. **(B)** Profiling the specificity of domains that recognize peptides that carry post-translational modifications. Phosphoamino acids such as phosphoserine (pS), phosphothreonine (pT), and phosphotyrosine (pY) can be incorporated into phage-displayed combinatorial peptide libraries or recombinant proteins. Affinity selections can be performed with these constructs to elucidate the specificity of protein interaction domains (top) as well as generate affinity reagents that recognize phosphoepitopes (bottom).

Two other recent publications of note describe the incorporation of an ncAA into the target of an affinity selection experiment (Figure 5A). Human interleukin-1β (IL-1β) and complement 5a (C5a) proteins were prepared in bacterial cells with *p*-benzoyl-L-phenylalanine utilizing GCE and used to affinity select a phage-display scFv library (Chen et al., 2020). By cross-linking virions to the targets with ultraviolet (UV) irradiation and washing away non-covalently bound virions with pH 2.0 glycine, the bound virions were selectively recovered by trypsin digestion. Over one-third of the recovered scFvs could bind to the targets without cross-linking and their affinities could be improved by mutagenesis. Thus, GCE can be used to steer production of antibodies to epitopes of interest. In a related approach, the 3-nitrotyrosine (nitroTyr) modified form (nY133) of the 14-3-3 signaling protein was used to immunize alpacas and two nanobodies that recognize this post-translational modification at position 133 were recovered by phage-display (Van Fossen et al., 2022).

## Perspectives

Several challenges and opportunities remain for optimizing GCE for phage-display. First, in order to incorporate a particular ncAA, the ncAA or its precursor must be supplied to the culture medium for it to cross the bacterial cell membrane where it can cross the cell membrane and be used by an aaRS to charge the suppressor tRNA. This challenge may be solved through the use of mutated strains, the expression of exogenous transporters, and the design of gene clusters capable of cellular synthesis of certain ncAAs. Second the use of bacterial strains that lack release factor 1 (RF-1) (Lajoie et al., 2013; Mukai et al., 2015; Fredens et al., 2019) will suppress TAG codons more efficiently.

One might ask, what are some possible experiments that can be accomplished by combining GCE and phage-display? An example that comes to mind is the production of phage-displayed combinatorial peptide libraries that carry phosphoserine (Park et al., 2011; Zhu et al., 2021), phosphothreonine (Zhang et al., 2017), or phosphotyrosine (Hoppmann et al., 2017; Luo et al., 2017). Such libraries could then be used to define the specificity of naturally occurring protein interaction modules that recognize phosphoepitopes (Yaffe and Elia, 2001; Liang and Van Doren, 2008; Marasco and Carlomagno, 2020), such as Src Homology 2 (SH2), phosphotyrosine binding domains (PTB), 14-3-3 proteins, BRCA1 C-terminus (BRCT) domains, and Forkhead-associated (FHA) domains (Figure 5B). The same libraries could be used to define the specificity of antibodies generated to phosphopeptides (Mandell, 2003). It might even be possible to construct libraries that display more than one post-translational modification (Qin and Liu, 2022) to elucidate the specificity of those domains that recognize two post-translational modifications simultaneously. This general approach should be applicable to defining the specificity of cellular proteins that bind other post-translational modifications. Another application might be to generate recombinant proteins that bear a phosphorylated amino acid at a position found in cellular proteins and then generate recombinant affinity reagents that recognize the folded, phosphorylated target (Figure 5B). Thus, it may even be possible to use this general approach to generate recombinant antibodies to many of the site-specific post-translational modifications of interest to cell biologists and biochemists. Finally, it is likely that chemically-and genetically-modified virions will prove beneficial in such emerging applications of phage in drug delivery (Karimi et al., 2016), immune-oncology (Foglizzo and Marchiò, 2021), synthetic biology (Lemire et al., 2018), tissue regeneration (Cao et al., 2019), and vaccines (Henry et al., 2015; González-Mora et al., 2020).

## Author contributions

AG and GA prepared the figures. All the authors contributed to the writing or editing the manuscript.
